# Single‐Cell Simultaneous Metabolome and Transcriptome Profiling Revealing Metabolite‐Gene Correlation Network

**DOI:** 10.1002/advs.202411276

**Published:** 2024-12-04

**Authors:** Xiying Mao, Dandan Xia, Miao Xu, Yan Gao, Le Tong, Chen Lu, Weiqi Li, Runmin Xie, Qinghuai Liu, Dechen Jiang, Songtao Yuan

**Affiliations:** ^1^ Department of Ophthalmology The First Affiliated Hospital of Nanjing Medical University Nanjing 210029 P. R. China; ^2^ The State Key Lab of Analytical Chemistry for Life Science Chemistry and Biomedicine Innovation Center (ChemBIC) School of Chemistry and Chemical Engineering Nanjing University Nanjing 210093 P. R. China

**Keywords:** functional metabolomics, mass spectroscopy, multi‐omics, RNA sequencing, single‐cell

## Abstract

Metabolic studies at the single cell level can directly define the cellular phenotype closest to physiological or disease states. However, the current single cell metabolome (SCM) study using mass spectroscopy has difficulty giving a complete view of the metabolic activity in the cell, and the prediction of the metabolism‐phenotype relationship is limited by the potential inconsistency between transcriptomic and metabolic levels. Here, the single‐cell simultaneous metabolome and transcriptome profiling method (scMeT‐seq) is developed at one single cell, based on sub‐picoliter sampling from the cell for the initial metabolome profiling followed by single cell transcriptome sequencing. This design not only provides sufficient cytoplasm for SCM but also nicely keeps the cellular viability for the accurate transcriptomic analysis in the same cell. Integrative analysis of scMeT‐seq reveals both dynamical and cell state‐specific associations between metabolome and transcriptome in the macrophages with defined metabolic perturbations. Moreover, metabolite signatures are mapped to the single‐cell trajectory and gene correlation network of macrophage transition, which allows the unsupervised functional interpretation of metabolome. Thus, the established scMeT‐seq should lead to a new perspective in metabolic research by transforming metabolomics from a metabolite snapshot to a functional approach.

## Graphical abstract

An approach is demonstrated to simultaneously profile metabolome and transcriptome in one single macrophage. The direct and real‐time relationship between the metabolome and transcriptome displays the properties of cellular heterogeneity and intracellular dynamicity. Furthermore, the integrative correlation network of metabolome and transcriptome enables the direct inference of metabolites linking with transcriptional modules, facilitating functional annotations and biological perspectives of metabolomics.

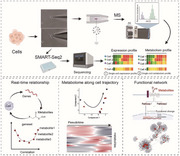

## 1 Introduction

Single‐cell omics technologies, such as single‐cell genome, epigenome, transcriptome, proteome, and metabolome, enable a deep depiction of the landscape of cellular molecular biological processes. They facilitate to describe the complete procedure of the “central dogma” and deconstructs the molecular interaction mechanism and targets from macromolecules (nucleic acids, proteins) to small molecules (metabolites), uncovering the nature of cellular heterogeneity. Moreover, single‐cell omics analysis has had a transformative impact on cell lineage tracing, tissue‐specific and cell‐specific atlas production, tumor immunology, and cancer genetics research. To achieve the comprehensive characterization of single cell, single cell multi‐omics analysis by combining different omics technologies is emerging that revolutionizes molecular cell biology research. Considering the huge heterogeneity of single cells, multi‐omics analysis needs to be conducted at one cell.^[^ ^1^ ^]^ However, due to limited sensitivity in single cell proteome and metabolome using mass spectroscopy, incompatible extraction buffers, and sample pretreatment protocols, it is extremely difficult to achieve multiple omics analysis in one cell. More recently, the breakthrough was achieved by precise sample‐splitting of single mouse oocyte cells in the nanoliter range to complete simultaneous deep transcriptome and proteome profiling in a single cell.^[^ ^2^ ^]^ Despite this achievement offers the feasibility in the co‐characterization of transcriptome and proteome in one cell, relatively large oocytes are used which restricts the further application for most mammalian cells.

Similarly, the coupling of metabolome and transcriptome from the same single‐cell individual is urgently demanded. Metabolome is the last omics layer that pools effects from epigenome, genome, and transcriptome, which directly defines the cellular phenotype closest to physiological or disease states. Thus, metabolic analysis provides meaningful insight into biology, which can often not be captured on other omics layers. Electrospray ionization MS (ESI‐MS), coupled with a capillary‐based technique to collect the raw cytoplasm from single cells, is leading the way for single cell metabolome (SCM).^[^ ^3^, ^4^ ^]^ However, the data bias caused by the effect on cell metabolism and viability during sample preparation cannot be ignored. Moreover, SCM only gives a snapshot of intermediates and products of cellular metabolism without the clue of metabolic flux and function, limiting the interpretation of metabolic mechanisms in biology.

To overcome the barrier of biological interpretation of metabolome, metabolic modeling by single‐cell RNA transcriptomics (scRNA‐seq) offers another approach for SCM. The use of prior knowledge from curated metabolic pathways enables the prediction of metabolism‐phenotype relationship.^[^ ^5^ ^]^ This information provides mechanistic insights into cellular metabolism that is hard to convey by measuring metabolite concentrations. Nevertheless, the transcriptome‐based SCM is limited by the potential inconsistency between transcriptomic level and metabolic level due to, for instance, posttranscriptional modification. In addition, the tradeoff between enzyme activity and metabolite concentration commonly exists in environment alterations, especially metabolic perturbation, to maintain homeostasis.^[^ ^6^ ^]^ Such circumstance leads to different relationships, even reverse correlation, between metabolic reaction and enzyme expression. Therefore, to closely recapitulate the metabolic state and function, the development of single cell metabolome and transcriptome omics from the same single‐cell individual is important for single cell study. Unfortunately, the incorporation of single cell metabolome (SCM) into multi‐omics analysis is more challenging because the metabolic processes are highly dynamic and need real‐time analysis.

In this study, we for the first time developed a single‐cell simultaneous metabolome and transcriptome profiling method (scMeT‐seq) to give a complete view of cellular activity. Different from the traditionally used micro‐capillary for single cell sampling, a nano‐capillary with an opening of 300 nm was inserted into a single living cell that could sort a tiny amount of cytoplasm immediately for the metabolic analysis. The process will keep most of the cytoplasm inside the cell, remaining its activity for the following transcriptome profiling. We validated the reliability, robustness, and biological interpretability of scMeT‐seq in vitro primary murine macrophages. It allows the identification of the undiscovered relationships between transcriptome and metabolome in cell state transition. Moreover, the establishment of a metabolite‐gene correlation network shows considerable potential to predict the biological function of metabolites, which leads to the discovery of novel biomarkers and therapeutic targets in biomedical research.

## 2 Results

### 2.1 The Design of scMeT‐Seq

**Figure 1:**
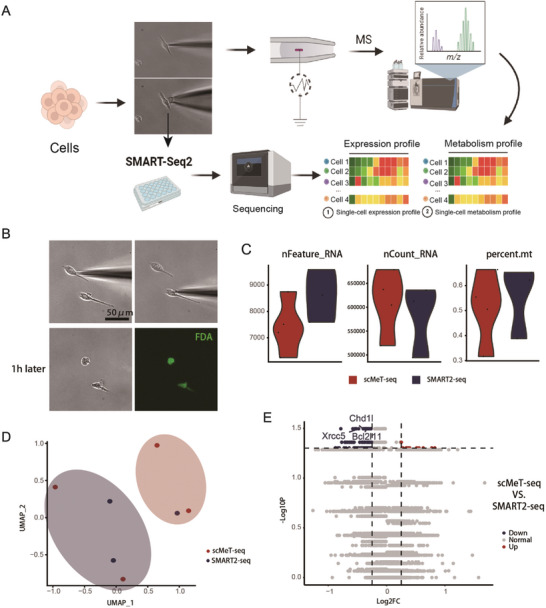
scMeT‐seq combines metabolome profiling by sub‐picoliter sampling with SMART‐seq2. A) Schematic diagram showing the procedure of scMeT‐seq in which a nano‐capillary with the opening of 300 nm was inserted into a single living cell to sort a tiny amount of cytoplasm for the metabolic analysis and the cell was immediately aspirated into a 9 µm opening capillary containing lysis buffer which was for the subsequent conventional SMART‐seq2 procedure. B) Representative images of the scMeT‐seq sampling procedure. One hour after sampling, the cell preserved viability as evidenced by cell morphology and FDA staining. Scale bar, 50 µm. C) Quality control of scMeT‐seq and the whole‐cell SMART‐seq2 applied on primary mouse macrophages based on the parameters that are listed above each panel. nFeature_RNA, number of detected genes; nCount_RNA, total count of all genes; percent.mt, percentage of counts from mitochondrial genes. D) UMAP visualization of the single‐cell transcriptome data from scMeT‐seq (n = 4) and the whole‐cell SMART‐seq2 (n = 3). E) Volcano plot of DEGs of single‐cell transcriptomic data between scMeT‐seq and whole‐cell SMART‐seq2.

Considering highly dynamic metabolic processes in a single living cell, single cell metabolome analysis must be performed first followed by single cell transcriptome profiling. Therefore, the key in the established scMeT‐seq is to perfectly balance the sorting amounts from one cell in metabolome and transcriptome analysis. That means, sufficient cellular cytoplasm for single cell metabolome should be guaranteed, but also the cytoplasm should be maximally retained for the following transcriptome analysis. This dilemma is solved in our study by using a capillary with a nanometer‐sized opening. The flow chart for scMeT‐seq is illustrated in **Figure** **1**A. Upon the position of a 300 nm opening nano‐capillary into the living model cell (primary mouse peritoneal macrophages), the punctured area is tiny as compared with the micrometer‐sized cell surface (Figure S1A, Supporting Information). The post‐operative cells preserved their viability as evidenced by cell morphology and division (Figure 1B, Figure S1B, Supporting Information), suggesting a minor interruption of cellular activity during this sampling process. Accordingly, the reservation of cellular state permits the following single cell transcriptome analysis with accuracy. Despite only sub‐picoliter cytoplasm is sorted, the mass spectroscopy with more than 1000 ion peaks was successfully obtained due to high ionization efficiency in nano‐opening. About 100 intracellular metabolites were identified among them, involving multiple metabolic pathways, such as glyoxylate and dicarboxylate metabolism, pyrimidine metabolism, citrate cycle, pyruvate metabolism, etc (Table S1, Supporting Information). The coverage of most metabolites exhibits the feasibility of single cell metabolome analysis using our established nano‐capillary based technique.

Using nanocapillary‐based sampling, a small amount of cytoplasm in a small region was extracted for mass spectrometry analysis. Given the heterogeneous nature of the cytoplasmic solution, a “mother‐daughter cell model” was used to assess whether this heterogeneity affects experimental outcomes significantly. Following cell division, the two daughter cells of a single mother cell exhibit similar positioning and morphology (Figure S2A, Supporting Information). Theoretically, the cell states of these two daughter cells should be largely identical. In this experiment, each pair of daughter cells was sampled based on distinct positional criteria: the sampling location for the first daughter cell was set at the center of the cell (Group A), while the second daughter cell was sampled at the periphery of the cell (Group B). Both groups were subjected to metabolomic analysis. The results demonstrated substantial overlap between the two groups in the PCA plot (Figure S2B, Supporting Information). In addition, the metabolomic profiles of all six pairs of daughter cells were clustered on the same terminal branches in hierarchical clustering dendrogram analysis (Figure S2C, Supporting Information). Likewise, the heatmap distribution of these metabolites revealed no significant differences between groups, with each pair of daughter cells clustering together (Figure S2D, Supporting Information). These findings suggest that the variation introduced by sampling heterogeneity is less pronounced than the inherent variability within cells of the same type.

Immediately after the sorting from the target cell, another capillary with an opening size of 9 µm was filled with lysis buffer, approaching the cell with the control of a micromanipulator (Figure 1A). As shown in the video (Movie S1, Supporting Information), upon contact between the capillary and the cell surface, an air pump device is activated to apply negative pressure. In the presence of triton in the lysis buffer, the cells become softened and can be readily aspirated into the capillary, which was transferred to an EP tube for subsequent single‐cell transcriptomics analysis using the conventional SMART‐seq2 procedure. An average of 7424 genes were identified at a sequencing depth of ≈0.6 million reads per cell. The fraction of mitochondrial genes was less than 10% (Figure 1C, Figure S3A,B, Supporting Information). These results demonstrated comparable quality to that obtained with the stand‐alone SMART‐seq2. To rule out the potential influence of cytoplasmic extraction on the transcriptome, we compared the gene expression profile of scMeT‐seq with that generated by the whole‐cell SMART‐seq2. Using the same cell type and culture conditions, we found similar quality control parameters. Principal component (PC) and differentially expressed gene (DEG) analysis revealed indistinguishable expression profiles between the two techniques (Figure 1D,E, Data S1, Supporting Information). Furthermore, we benchmarked the DEGs of lactate‐treated macrophage from scMeT‐seq to that from bulk RNA‐seq. scMeT‐seq‐based upregulated genes were 50% overlapped with that from the data obtained from bulk samples (Figure S3C, Supporting Information). Taken together, these results constitute the methodological foundation of scMeT‐seq for the simultaneous single‐cell profiling of metabolome and transcriptome.

### 2.2 Single‐Cell Consistency Between Transcriptome and Metabolome

**Figure 2:**
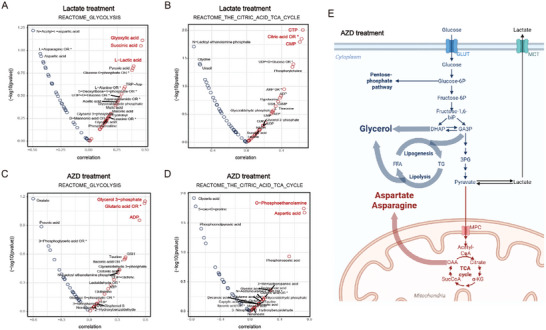
Correlation analysis between transcriptomics‐assisted scoring of metabolic pathways and metabolites. A) Volcano plots of Spearman correlation coefficient between GSVA scoring of REACTOME GLYCOLYSIS pathway and the metabolites in scMeT‐seq data of lactate‐treated primary macrophages. B) Volcano plots of Spearman correlation coefficient between GSVA scoring of REACTOME_THE_CITRIC_ACID_TCA_CYCLE_AND_RESPIRATORY_ELECTRON_TRANSPORT pathway and detected metabolites in scMeT‐seq data of lactate‐treated primary macrophages. C) Volcano plots of Spearman correlation coefficient between GSVA scoring of REACTOME GLYCOLYSIS pathway and detected metabolites in scMeT‐seq data of AZD3965‐treated primary macrophages. D) Volcano plots of Spearman correlation coefficient between GSVA scoring of REACTOME_THE_CITRIC_ACID_TCA_CYCLE_AND_RESPIRATORY_ELECTRON_TRANSPORT pathway and detected metabolites in scMeT‐seq data of AZD3965‐treated primary macrophages. E) Schematic representation of the shift of metabolic flux from glycose catabolic pathway to biomass synthesis, such as lipid deposition and aspartate synthesis, in the treatment of AZD3965.

To assess the sensitivity of scMeT‐seq to capture cellular metabolic dynamics, primary macrophages were treated with lactate and the MCT1 inhibitor AZD3965, respectively. Lactate serves as a primary carbon fuel source and exerts a complicated effect on metabolic pathways.^[^ ^7^ ^]^ AZD3965 inhibits the export of lactate, thereby contributing to the passive intracellular accumulation of lactate.^[^ ^8^ ^]^ Therefore, these two treatments have distinct effects on both intracellular metabolism and macrophage polarization. Based on the different treatments on macrophages, the relationships between transcriptomic and metabolomic dynamics were investigated using the established scMeT‐seq method. In detail, gene set variation analysis (GSVA) was applied to the transcriptomic data of scMeT‐seq to predict the metabolic pathways of single cells. Correlation analysis was then performed to examine the cell‐wise covariations between the GSVA scoring of metabolic pathways and the concentration of metabolites. We focused on the pathways related to glucose metabolism in the lactate‐treated group and found a consistent pattern of change in the transcriptome and metabolome. The predicted glycolytic activity was positively correlated with L‐lactic acid, pyruvic acid, and glucose‐6‐phosphate (**Figure** **2**A). Meanwhile, the TCA cycle pathway was positively correlated with citric acid and nucleotides (CTP, CMP) (Figure 2B).

However, the consistency between transcriptome and metabolome in glucose metabolism was diminished in AZD3965‐treated group. Glycolytic intermediates, such as phosphoenolpyruvic acid, pyruvic acid, and phosphoglyceric acid, were negatively correlated with the predicted pathways (Figure 2C,D). Otherwise, glycerol 3‐phosphate and glutaric acid were positively correlated with the expression of the glycolytic gene set, while aspartic acid and O‐phosphoethanolamine were accumulated along with predicted TCA pathway activity (Figure 2C,D). The inconsistency may be due to the metabolic stress caused by passive lactate accumulation in AZD3965 treatment,^[^ ^9^ ^]^ in which the metabolic flux was shifted from the glucose catabolic pathway to biomass synthesis, such as lipid deposition and aspartate synthesis (Figure 2E).^[^ ^10^, ^11^, ^12^, ^13^ ^]^ Therefore, in the case of metabolic imbalance, the inconsistency between transcriptome and metabolome may lead to a breakdown of the analytical premise that the transcripts can predict metabolite quantities, which highlights the importance of multi‐omics metabolic profiling.

### 2.3 Unraveling Hysteretic and Stepwise Changes of Metabolome During Transcriptomic Transition

**Figure 3:**
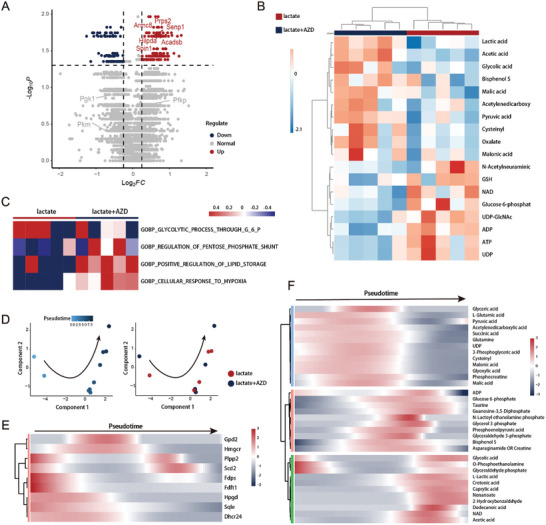
Single‐cell dynamics of transcriptomics and metabolomics in the treatment of AZD3965. A) Volcano plots showing *Prps2*, *Armc8*, *Hilpda*, *Acadsb*, and *Spin1* among the significantly upregulated genes in AZD3965 treatment. Red and blue dots represent genes with a significant difference. B) Heatmap of differential single‐cell metabolites in AZD3965 treatment (lactate+AZD) and control (lactate). C) Heatmap showing GSVA enrichment scores for respective GO biological processes in each cell with (lactate+AZD) or without AZD3965 treatment (lactate). D) Pseudotime trajectory based on the transcriptomic data of scMeT‐seq showing the distribution of single macrophages in the treatment of AZD3965. Pseudotime and different treatments are shown in different colors, respectively. E) Heatmap showing the expression of lipid metabolism‐associated genes along the pseudotime shown in Figure 3D. F) Heatmap showing the differential metabolites along the pseudotime shown in Figure 3D.

Intergroup comparison in transcriptome by scMeT‐seq after AZD3965 treatment showed that genes related to nucleotide biosynthesis (*Prps2*, *Armc8*) and lipid metabolism (*Hilpda*, *Acadsb*, *Spin1*) were upregulated (**Figure** **3**A, Data S1, Supporting Information). However, no evidence of corresponding metabolites was observed in the intergroup comparison of the metabolome (Figure 3B). In the differential metabolite analysis, glycolytic intermediate glucose‐6‐phosphate was downregulated in AZD3965 treatment, but glycolysis‐related genes did not show significant change (Figure 3B,C, Figure S4A,B, Supporting Information). For this inconsistency shown in the between‐group comparisons, we hypothesized that it could be attributed to the heterogeneity of metabolome and transcriptome. The heterogeneity of the transcriptome has currently been interpreted with the development of single‐cell transcriptomic sequencing, but the metabolomic heterogeneity and its correlations with the single‐cell transcriptome remain elusive.

To further dissect the multi‐omics heterogeneity during the cell state transition, cells from both groups were incorporated and subjected to single‐cell pseudotime analysis. Pseudotime trajectory was constructed based on the multi‐omics status of single cells (Figure 3D, Figure S5, Supporting Information). At the transcriptome level, genes functioning in lipid biosynthesis were expressed from the start of pseudotime (Figure 3E). Subsequently, the anti‐cancer function of AZD3965 was manifested along with the upregulation of inflammatory signaling pathways and apoptotic process at the end of pseudotime (Figure S5, Supporting Information). However, the changes at the metabolomic level appeared to be more complicated. The level of TCA cycle intermediates was downregulated along the pseudotime of AZD3965 treatment. The glycolytic intermediates exhibited transient upregulation, among which glycerol 3‐phosphate serves as the shunt in the regulation of glucose metabolism and lipogenesis. At the endpoint of pseudotime, lipid synthesis‐related intermediates began to accumulate and eventually achieved consistency with transcriptomic change (Figure 3F). The above results indicate that the expression of enzyme‐encoding genes determines the direction of metabolomic changes and leads to the establishment of metabolomic homeostasis in a lagging and progressive manner. Our established scMeT‐seq delineates for the first time the asynchronous biology of metabolomic dynamics in the response to transcriptomic transition.

### 2.4 Deciphering Cell State‐Resolved Metabolomics

**Figure 4:**
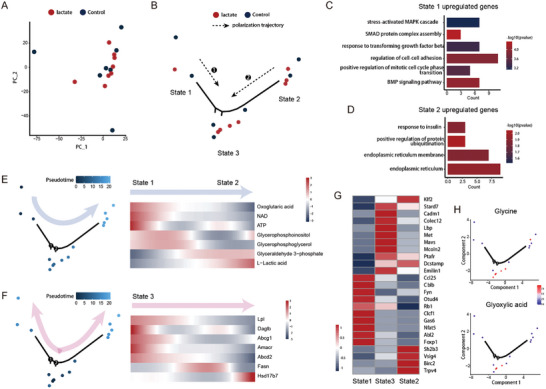
Deciphering macrophage cell state‐resolved metabolomics with lactate treatment. A) PCA visualization of lactate‐treated (n = 8) and control (n = 7) primary macrophages, colored by different treatments. B) Pseudotime trajectory based on the transcriptomic data of scMeT‐seq showing the distribution of single macrophages in the treatment of lactate. Different treatments are shown in different colors. C) GO enrichment of upregulated genes in State 1 shown in Data S2 (Supporting Information). D) GO enrichment of upregulated genes in State 2 shown in Data S2 (Supporting Information). E) Heatmap showing the characteristic metabolites along the indicated pseudotime shown in the left panel. F) Heatmap showing the expression of lipid metabolism‐associated genes along the indicated pseudotime shown in the left panel. G) Heatmap showing the expression of immune activity‐associated genes among the three states. H) The normalized intensities of glycine and glyoxylic acid on the pseudotime trajectory shown in Figure 4B.

The transition of cell state is generally not unidirectional even within the same treatment assay. Metabolomic changes accompanying the multidirectional cell state transition can currently only be inferred by transcriptome‐based single‐cell metabolic analysis, whereas transcriptomic change of corresponding metabolic enzymes is often insensitive and deviates from the real metabolic flux. The direct analysis of metabolomics, on the other hand, is limited by its cell identity‐ or cell state‐resolving power. Using scMeT‐seq for the analysis of the lactate‐treated primary macrophage, the lactate‐treated cells were observed to show two heterogeneous cell states that eventually converged into a new state along pseudotime (**Figure** **4**A,B).

The molecular difference between the two putative polarization trajectories was further explored (Data S2, Supporting Information). Genes related to TGFβ and BMP pathway were enriched in one of the cell trajectories, which were accompanied by the increased levels of oxoglutaric acid, NAD, and ATP (Figure 4C,E). The content of glycerophospholipids, such as glycerophosphoinositol (PI), glycerophosphoglycerol (PG), were also increased. As the most abundant lipid class present in cell membranes, PI and PG were especially important for the mitochondrial membrane, which may thus be expected to be the indicator for OXPHOS.^[^ ^14^ ^]^ Along the other trajectory, GO terms, such as “positive regulation of protein ubiquitination”, “endoplasmic reticulum” and “response to insulin”, were present (Figure 4D). In terms of metabolomics, glycolysis‐related metabolites glyceraldehyde 3‐phosphate and lactic acid were enriched (Figure 4E). The above results suggest that distinct metabolic states occurred on respective polarization trajectories within the same environment. It is noted that no significant difference was observed in OXPHOS and glycolytic pathways between the two cell trajectories when analyzing only the transcriptomic data, again indicating the defective mapping relationship between transcriptome and metabolome (Figure S6B,C, Supporting Information). Although the phenomenon of metabolic coexistence has been inferred in previous single‐cell transcriptomic studies, scMeT‐seq allows the acquisition of such cell state‐corresponding metabolomes, providing the direct read‐out of the metabolomic landscape.

As the progression of pseudotime, the two cell states gradually converged into a cell cluster with altered profiles of transcriptome and metabolome. At the transcriptomic level, GO term “negative regulation of macrophage derived foam cell differentiation” was significantly enriched in this cluster (Figure S6A, Supporting Information). Genes related to lipolysis (*Lpl*, *Daglb*), export (*Abcg1*, *Abca5*), and oxidation (*Amacr*, *Abcd2*) were specifically expressed, while lipid synthesis genes expressed at the two branches of cell trajectories, e.g., *Fasn*, *Hsd17b7*, were downregulated in expression (Figure 4F). The metabolome in this cluster further showed that the OXPHOS‐related metabolites oxoglutaric acid, NAD, and ATP remained at high levels, whereas the level of lactic acid decreased (Figure S6C, Supporting Information). The information of two omics complementarily revealed the predominant role of lipid oxidation in energy supply at this stage. Meanwhile, the expression profiles of immune reaction‐related genes also suggested that macrophages were further activated to the suppressive phenotype (M2‐like) that relies mainly on OXPHOS (Figure 4G).

Interestingly, we found a significant increase in the levels of glycine and glycine derivative glyoxylic acid in the terminal state (Figure 4H). The level of glycine has been observed in metabolic disorders associated with insulin resistance, which suggests the potential connection between glycine metabolism and lipid metabolism.^[^ ^15^ ^]^ Taken together, the combination of transcriptome and metabolome provides a more comprehensive inference of metabolic landscape than a single‐omics study: lactate‐treated macrophages select lipid oxidation for energy supply while adaptively diverting glycolysis to amino acid metabolism. Furthermore, the treatment of glycine was previously demonstrated to induce the anti‐inflammatory polarization of macrophage, consistent with the above findings in the cell state‐resolved metabolic analysis.^[^ ^16^ ^]^ Thus, we infer that scMeT‐seq could provide a reference coordinate system for functional phenotyping of metabolomics.

### 2.5 Establishing Predicted Link Between Metabolites and Biological Functions

**Figure 5:**
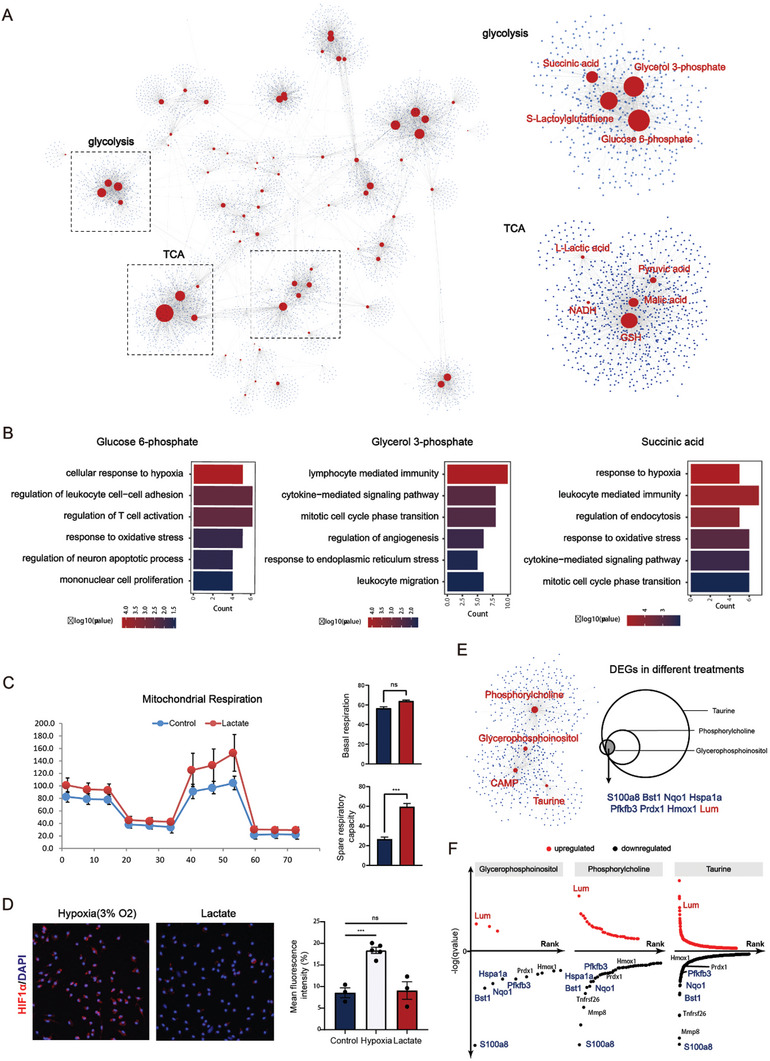
Metabolite‐gene correlation network analysis predicts metabolic functionality. A) The metabolite‐gene correlation network shows different gene modules centered on individual metabolites. Two gene modules are indicated in the association with glycolysis and TCA cycle, respectively. B) GO enrichment of genes correlated with Glucose 6‐phosphate, Glycerol 3‐phosphate, and Succinic acid, respectively, by using the Spearman method. C) Seahorse analysis of mitochondrial respiration of primary macrophages with indicated treatments for 72h. Bar graph showing the differential levels of basal respiration and spare respiratory capacity. D) Representative immunofluorescent images and corresponding quantified mean fluorescence intensity of HIF1a expression in primary macrophages after indicated treatments for 72h. E) The colocalization of metabolites cAMP, glycerophosphoinositol, phosphorylcholine, and taurine within one gene module. F) Dot plot showing the ranking of DEGs of indicated treatments based on *q*‐value.

Metabolite profiling provides direct information on metabolic phenotypes and indirect functional information, based on grouping approaches for whole profiles. However, the functional genomics of individual metabolites remains a major challenge in the interpretation of metabolomic data. Our scMeT‐seq offers an approach to overcome+ the limitation of grouping comparisons and enables the identification of the relationship between the highly dynamic metabolites and the corresponding transcriptome. We integrated the expression matrix of the transcriptome with the quantification matrix of metabolome and conducted the co‐expression network analysis of metabolites and genes. In the constructed network, metabolites were placed in the hub and centered by the correlated genes (**Figure** **5**A, Data S3, Supporting Information). The glycolytic intermediates glucose 6‐phosphate, glycerol 3‐phosphate, and S‐Lactoylglutathione were found to be present within a gene module, which was correlated with biological pathways such as hypoxia‐induced pathway, leukocyte activation, and proliferation (Figure 5B, Data S4, Supporting Information). Unexpectedly, succinic acid, a TCA cycle intermediate, was also clustered with the glycolytic intermediates. Recently, a novel role for succinate outside metabolism has been uncovered to stabilize HIF 1α in activated macrophages.^[^ ^17^ ^]^ In contrast, L‐lactic acid, previously considered to be the end‐product of glycolysis, was otherwise clustered with TCA cycle intermediates (Pyruvic acid, Malic acid, and GSH). In vitro, treatment of primary macrophage with L‐lactic acid resulted in increased mitochondrial oxidation without evidence of hypoxia (Figure 5C,D, Figure S7A, Supporting Information). Taken together, we postulate that the single‐cell metabolite‐gene correlation network may reflect the biological function of metabolites in an unbiased manner, beyond their metabolic aspect.

To demonstrate the predictive role of metabolite function by scMeT‐seq, we selected a gene module containing metabolites with less‐studied biological functions. For instance, cAMP, glycerophosphoinositol, phosphorylcholine, and taurine colocalized within the same gene module. cAMP is a well‐characterized cellular second messenger with a defined role in the resolution of inflammation,^[^ ^18^ ^]^ whereas the effect of other metabolites on macrophage activation remains elusive. To elucidate the effect of these metabolites on macrophages, lactate‐pretreated macrophages were treated with glycerophosphoinositol, phosphorylcholine, and taurine, respectively (Data S5, Supporting Information). DEGs from glycerophosphoinositol and phosphorylcholine treatment largely overlapped with those from taurine treatment (Figure 5E, Figure S7B, Supporting Information). *S100a8* was the most significantly downregulated gene among the DEGs of the three treatments, indicating an anti‐inflammatory effect in activated macrophages.^[^ ^19^ ^]^ The expression of antioxidant genes *Ngo1*, *Prdx1*, and *Hmox1* were collectively downregulated, consistent with the known function of cAMP in promoting mitochondrial dysfunction and oxidative stress (Figure 5F).^[^ ^20^ ^]^ Furthermore, reduced expression of glycolysis‐related genes, such as *Pfkfb3*, *Pkm*, and *Ldha*, was observed compared to the control macrophages. These results suggest that the metabolic transition may be associated with the altered polarization of macrophage.^[^ ^21^ ^]^ Consistent with the above findings from bulk sequencing, the gene network generated from scMeT‐seq showed complete segregation from the glycolysis‐related gene network, but a relatively proximal distance to the TCA‐related gene network (Figure 5A). Taken together, the power of scMeT‐seq is highlighted to generate a testable hypothesis for metabolic functionality.

## 3 Discussion

The investigation of metabolic heterogeneity has grown considerable attractions, which calls for single‐cell‐resolution measurements of metabolic dynamics. However, the approach that bridges the gap between metabolomics and metabolic phenotype at single‐cell resolution is lacking. Here, we reported scMeT‐seq, a method that simultaneously profiles the metabolome and transcriptome in single cells. The data offers crucial insight into the correlations between metabolome and transcriptome. Furthermore, it provides an unprecedented opportunity to investigate the single‐cell metabolite‐gene regulatory network, which bypasses the limitation of comparisons between experimental and control samples that were commonly used in bulk metabolomics analysis. The establishment of metabolite‐gene network may exert a broader implication in linking metabolites to their functions and pathologies, which facilitates future experimental design and the development of druggable small molecules.

Currently, single‐cell metabolic profiling is mainly based on transcriptomic analysis. mRNA abundance was used to predict metabolic enzyme activity and flux via a range of approaches, such as pathway‐based analysis and constraint‐based modeling.^[^ ^4^ ^]^ The mapping from gene expression to metabolism is based on the assumption in metabolism is in a steady‐state versus the related genes that come from prior biological knowledge. However, as revealed in scMeT‐seq, a passive mechanism may take place to maintain metabolic homeostasis upon metabolic perturbation. Previously, metabolomic change was thought to be instantaneous and transient. In this study, by parallelly comparing transcriptome and metabolome at single‐cell pseudotemporal resolution, we uncovered a lagging and stepwise nature in the establishment of metabolomic homeostasis, which leads to an inconsistent relationship between transcriptome and metabolome. Under such circumstances, gene expression fails to reflect the real‐time composition of metabolites. Therefore, the simultaneous information of two omics is indispensable and reciprocal for deciphering a comprehensive landscape of single‐cell metabolism.

Integrative analysis of transcriptome and metabolome conventionally relies on the bulk sample being split in two and subjected to transcriptomic sequencing and metabolite quantification, respectively. However, this approach does not reveal a true correspondence. In addition, intra‐sample heterogeneity is not taken into account, leading to average information that introduces a significant bias in the biological interpretation. Recently, scSpaMet has been developed to incorporate untargeted spatial metabolomics and targeted multiplexed protein imaging in the same tissue, enabling the detection of cell type‐resolved metabolome. Moreover, metabolomic changes along B cell differentiation trajectories could be profiled.^[^ ^22^ ^]^ However, the single‐cell pseudotime construction was based on the prior knowledge of cell identity. When the process of cell differentiation is unknown or the difference in cell state cannot be distinguished with specific markers, targeted multiplexed protein detection showed limited values. In our study, by combining untargeted metabolomics and RNA sequencing at single‐cell resolution, thousands of genes can be obtained for the definition of cell state and function and contribute to the real single‐cell integration of transcriptomic and metabolomic omics, unveiling the cell state‐resolved metabolomics and genome‐wide crosstalk with metabolism.

One major limitation of scMeT‐seq is the low throughput in the implementation of nano‐capillary for cellular sampling, as the sampling is manual. A robotic arm can be utilized in combination with a plate‐based single‐cell scRNA‐seq methodology, which can theoretically be scaled up to hundreds of cells per day. In addition, subject to an extremely low amount of sorted cytoplasm and restricted ionization efficiency, the detected number of metabolites was far less than the detected genes, which overshadows the power of metabolites in the construction of a correlation network. Second, single‐cell bioinformatics of metabolome combined with transcriptome is still in its infancy. Conventionally, gene and metabolite enrichment analysis in tandem, termed sequential analysis, has been performed to analyze the integrative datasets, which inevitably lose the interactive information. For integrative analysis, strategies are established in the integration of multi‐omics inputs into a single dataset and then canonical correlation analysis is widely applied. However, it is criticized that the analysis is performed in a univariate way and ignores the dependence between metabolites.^[^ ^23^ ^]^ To achieve a global view of the metabolic network, the knowledge of the topology and stoichiometry of the metabolic network can be translated into mathematical models and allows a structural analysis of the metabolic network.^[^ ^24^, ^25^ ^]^ Currently, scRNA‐seq has been used as input for this approach, but the simultaneous input of more omics, such as scMeT‐seq data, will limit the solution space of constraint‐based metabolic models with greater accuracy.

## 4 Conclusion

In summary, we have developed a novel method to detect simultaneous dynamics of metabolome and transcriptome at single‐cell resolution. The established scMeT‐seq fills an important yet empty niche in the field of SCM by linking the metabolic status with a biological phenotype of individual cells. Our approach has documented previously uncharacterized patterns of metabolomic and transcriptomic variations. The simultaneous depiction of cellular states has led to a more comprehensive understanding of metabolism–transcriptome crosstalk. The cross‐omics data will likely pave the way for the identification of novel mechanistic and therapeutic insight into human diseases.

## 5 Experimental Section

### Cell Culture and Treatment

Primary peritoneal macrophages were isolated from 6‐month‐old male mice C57BL/6. The mice were euthanized, and the peritoneal cavity was injected with 7 mL precooled PBS supplemented with 3% fetal bovine serum (FBS, SenBeiJia). The isolation media was drawn back 30 min after injection through a 10 mL syringe equipped with a 25 g needle. The isolation media was centrifuged, and erythrocytes were lysed by the addition of RBC lysis buffer (Beyotime). The erythrocyte‐free peritoneal cells were resuspended in DMEM media (Gibco) supplemented with 20% FBS, 1 mm L‐glutamine (Gibco), 1% penicillin‐streptomycin (Gibco) and incubated at 37 °C and 5% CO2 for 24 h. After 24 h incubation, non‐adherent cells were removed by repeated flushing of the flasks with culture medium to obtain pure peritoneal macrophages. For in vitro treatment, primary mouse macrophages were treatments with 10 mm lactate (Sigma–Aldrich), 300 nm AZD3965 (MedChemExpress), 100um glycerophosphoinositol (EchelonBioscience), 100um phosphorylcholine (MedChemExpress), or 80 mm Taurine (MedChemExpress) for 72 h. To evaluate the effects of hypoxia, cells were incubated in normoxic conditions (21% O2) or 3% O2.

### Procedures of the scMeT‐seq platform

Borosilicate glass capillary (BF100‐58‐10, o.d. 1.00 mm, i.d. 0.58 mm) was purchased from Sutter Instrument (CA, USA). The capillaries used in the experiments were drawn using P‐2000 laser puller (Sutter Instrument, CA, USA). The opening of the capillary for single‐cell metabolome analysis was ≈300 nm, for single‐cell transcriptome analysis, was ≈9 µm. The capillary was connected to an air pumping device and then fixed at the InjectMan4 micromanipulator (Eppendorf, Germany) coupled with an IX51 microscope (Olympus, Japan). First, 300 nm nanocapillary filled with 10 µL ultrapure water was fixed at the micromanipulator to sample cell components for single‐cell metabolome analysis, after sampling, the nanocapillary was removed for mass spectrometry analysis. Then 9 µm microcapillary filled with 6 µL lysis buffer (2.5 mM dNTP, 2.5µM dT30VN oligo, 0.1% Triton‐X‐100 and 4U Recombinant ribonuclease inhibitor) was fixed at the micromanipulator to extract the same intact cell for single‐cell transcriptome analysis, after the extraction, the lysis buffer mixed with an intact cell was transferred to an EP tube for subsequent analysis by the air pumping device.

### MS Analysis

The 300 nm nanocapillary mentioned above with a metal wire inside was placed 1.5 cm away from the ESI source of G6530B Q‐TOF mass spectrometer (Agilent, CA, USA). 3500 V voltage was set at the metal wire. Using negative ion acquisition mode. The drying temperature was 300 °C. The drying gas flow rate was 2.0 L min^−1^. The MS scan range was set as 50–1000 m/z and the scan speed was set as 1 scan s^−1^.

### Metabolite Annotation and Analysis

The MS raw data (. d) were imported into Agilent MassHunter Quantitative Analysis Software (B.06.00) for analysis. The m/z and intensity value (>500 counts) of selected scans were exported to perform metabolite annotation and analysis. The metabolites were annotated based on MS/MS fragmentation information or primary mass spectrometry accurate mass, which was matched with the Human Metabolome Database (HMDB; https://hmdb.ca/spectra/ms_ms/search). The mass deviation was set lower than 30 ppm. The processed metabolite data were input into MetaboAnalyst (https://www.metaboanalyst.ca/faces/home.xhtml) for statistical analysis.

### scRNA‐Seq Library Preparation

The scRNA‐seq library was constructed following a modified Smart‐seq2 protocol.^[^ ^26^ ^]^ In detail, 4ul lysed sample was incubated at 72 ℃ for 3 min. The first‐strand cDNA was reverse‐synthesized using oligo(dT) and template‐switching oligonucleotides. The synthesized first‐strand cDNAs were amplified by 20 cycles. After purification, 0.1 ng of cDNA was used for Nextera tagmentation (Illumina) and library construction. Sequencing was then performed on Illumina HiSeq 4000.

### scMeT‐seq Data Processing and Analysis

Single‐cell samples were consolidated and subjected to sequencing on the Illumina Novaseq 6000 platform, targeting an average of 3 million reads per library in a paired‐end 150‐bp format. The raw sequencing data underwent demultiplexing and were subsequently processed with the fastp software (version 0.20.0) for quality trimming. The quality‐trimmed reads were then aligned to the mouse reference genome mm10 using the HISAT2 aligner (version 2.1.0) with standard settings. The counts of reads per gene were quantified utilizing the StringTie package. The processed data matrix was imported into Seurat (version 4.0). The transcriptome matrix was taken as input and then scaled it before using dimensionality reduction techniques. Next, PCA was performed on the scaled data and the top principal components were selected to preserve biological variation and remove technical noise. The quality‐controlled metabolic matrices were added to the new assay and performed data standardization and normalization. A graph‐based clustering method was used to cluster macrophages. Gene sets were selected in GSEA (https://www.gsea‐msigdb.org/gsea/index.jsp) and the pathway activity of the cells was accessed using the GSVA method.^[^ ^27^ ^]^ The pathway matrix and the normalized metabolome matrix were combined, correlation between pathways and metabolites was calculated using the Spearman method, and the results were visualized by scatter plot in ggplot2. Based on the grouping information, the Wilcoxon rank sum test was performed using the FindMarkers function. Genes with |LogFC|>0.25 and *p* < 0.05 were considered as signature genes. This threshold was chosen to balance sensitivity and specificity in detecting biologically meaningful differences. Due to the small sample size in the study, employing multiple testing corrections would significantly reduce the statistical power. The results were visualized by volcano plots of ggplot2. The Monocle2 package was used to analyze macrophage trajectories. The transcriptome and metabolite matrices were imported into Monocle and the feature genes and metabolites were used to sort the pseudotemporal trajectories of the cells. The “DDRTree” was applied to reduce the space to two dimensions and the trajectories in the reduced dimensional space were visualized using the function “plot_cell_trajectory”. Cells were divided into three states based on the trajectories, and Gene Ontology (GO) enrichment analysis was performed for the differentially expressed genes of each state using the ClusterProfiler package. The GENIE3 (https://bioconductor.org/packages/release/bioc/html/GENIE3.html) package was used to analyze the metabolite‐gene covariance network. The merged matrix of bi‐omics was imported and functional enrichment analysis was performed for the genes in the three selected modules using the above software and visualized them by bar plot of ggplot2. The schematic was drawn by the BioRender Graphics Tool (http://BioRender.com).

### Immunofluorescence Staining

For immunofluorescence staining, cells were harvested at 72 h post different treatments. Macrophages were fixed with 4% PFA (Servicebio) at 4 °C for 15 min and then washed with PBS 3 times (5 min time^−1^). These cell‐culture treated coverslips were subsequently incubated with primary antibodies HIF1α (dilution: 1:200, Cat. # ab179483, Abcam) at 4 °C overnight, and corresponding fluorescence‐conjugated secondary antibodies (dilution: 1:1000, abcam) at room temperature for 1 h. Cell nuclei were stained with DAPI (Sigma–Aldrich). Fluorescence was observed using a Leica microscope (THUNDER DMi8). For cell viability assay, post‐operative cells were incubated with 10µM fluorescein diacetate (Sigma) for 5 min. The plate was then detected by a fluorescence microscope (Ex. 488 nm).

### Seahorse Extracellular Flux (XF) Analysis

Primary peritoneal macrophages were seeded into XF96 cell culture microplates (Seahorse Bioscience) and stimulated with lactate, AZD3965, or culture medium as a negative control for 72 h. OCRs and ECARs were quantified using the XF96 instrument (Seahorse Bioscience) according to the manufacturer's protocol. Specifically, the assay medium was unbuffered RPMI‐1640 supplemented with 10 mM glucose, 1 mM pyruvate, and 2 mM L‐glutamine, pH7.4. The measurements were performed at 4.5‐min intervals (30s mixing, 1 min recovery, 3 min measuring) for 1.5h. Baseline OCR and ECAR, and their response to the indicated compounds (2.5 µM oligomycin, 1.5 µM FCCP, 500 nM rotenone, 500 nM antimycin and 50 mM 2‐deoxyglucose (2‐DG)) were determined.

### Bulk RNA Sequencing and Analysis

Total RNA was extracted using TRIzol reagent (Invitrogen, CA, USA) according to the instructions, and RNA purity and quantification were determined using the NanoDrop 2000 spectrophotometer (Thermo Scientific, USA), and assessed using the Agilent 2100 Bioanalyzer (Agilent Technologies, Santa Clara, CA, USA). CA, USA) to assess RNA integrity. Transcriptome libraries were constructed using the VAHTS Universal V6 RNA‐seq Library Prep kit according to the instructions. Transcriptome sequencing and analysis were performed by OE Biotech Co., Ltd. (Shanghai, China). The libraries were sequenced using the llumina Novaseq 6000 sequencing platform, and 150 bp paired‐end reads were generated. fastp software was used to process the raw reads in fastq format, and clean reads were obtained after removing the low‐quality reads for subsequent data analysis. The clean reads were mapped to the mm10 using HISAT2. FPKM of each gene was calculated and the read counts of each gene were obtained by HTSeq‐count. Differentially expressed genes were analyzed using DESeq2 software, where genes that met the thresholds of *q*‐value < 0.05 were defined as DEGs.

### Statistical Analysis

Statistical analyses were performed with the GraphPad Prism (GraphPad Software). A *p*‐value of less than 0.05 was considered statistically significant. Comparisons between the two groups were performed using an unpaired Student's *t*‐test. Comparisons between multiple treatment groups and a control group were performed using one‐ or two‐way ANOVA with post hoc Tukey's multiple comparison test.

## Conflict of Interest

The authors declare no conflict of interest.

## Author Contributions

X.M., D.X., M.X. contributed equally to this work. X.M., D.J., and S.Y. designed the research. D.X., Y.G., L.T., C.L., and W.L. carried out experiments. X.M., D.X., M.X., and R.X. performed data analysis. X.M., Q.L., D.J., and S.Y. wrote the manuscript.

## Supporting information



Supporting Information

Supplemental Movie 1

Supporting Information

## Data Availability

The data that support the findings of this study are available from the corresponding author upon reasonable request.
