# Supplementary material for: Single‐Cell Simultaneous Metabolome and Transcriptome Profiling Revealing Metabolite‐Gene Correlation Network
Source: Adv Sci (Weinh). 2024 Dec 4;12(4):2411276. doi: 10.1002/advs.202411276 (PMC11775534; doi:10.1002/advs.202411276)
Supplement: Supplementary file 1 — Supporting Information [file ADVS-12-2411276-s002.docx]

Supporting Information

Single-Cell Simultaneous Metabolome and Transcriptome Profiling Revealing Metabolite-Gene Correlation Network

Xiying Mao, Dandan Xia, Miao Xu, Yan Gao, Le Tong, Chen Lu, Weiqi Li, Runmin Xie, Qinghuai Liu*, Dechen Jiang*, Songtao Yuan*

**Figure S1. scMeT-seq preserves the cellular viability for the subsequent single-cell transcriptomic sequencing.** (**A**) Representative images of a nano-capillary with the opening of 300 nm for sub-picoliter sampling and a 9 μm opening capillary for collecting single cells. (**B**) Representative images of a post-sampling cell undergoing cell division.

**Figure S2. The intra-locational differences of metabolome within individual cells.** (**A**) The two daughter cells of a single mother cell exhibit similar positioning and morphology. (**B**) Principal component analysis of metabolomic data from different positions of each cell pair. (**C**) Hierarchical clustering dendrogram analysis showing metabolomic profile of all 6 pairs of daughter cells are clustered on the same terminal branches. (**D**) Heatmap showing no obvious differences of metabolites between the pair of daughter cells.

**Figure S3. Quality control of scMeT-seq transcriptomic data.** (**A-B**) Quality control of scMeT-seq transcriptomic data applied on primary mouse macrophages with variable treatments. nFeature_RNA, number of detected genes; nCount_RNA, total count of all genes; percent.mt, percentage of counts from mitochondrial genes. (**C**) Venn plot showing 50% upregulated DEGs (16% downregulated DEGs) of lactate treatment from scMeT-seq overlapped with that from bulk RNA-seq.

**Figure S4. Principal component analysis and heatmap of metabolites in single cells from different treatments.** (**A**) Principal component analysis of scMeT-seq metabolomic data of macrophages in the treatment of lactate and lactate+AZD3965. (**B**) Heatmap analysis of all differential single-cell metabolites in the treatment of lactate and lactate+AZD3965. (**C**) Principal component analysis of scMeT-seq metabolomic data of macrophages in the control and lactate-treated group. (**D**) Heatmap analysis of all differential single-cell metabolites in the control and lactate-treated group. The names of metabolites denoted by * are shown in Table S1.

**Figure S5. Gene expression dynamics along the cell transition trajectory in the treatment of AZD3965.** Heatmap of the differential expressed genes along the pseudotime of cell transition in the treatment of AZD3965 (Left panel). GO enrichment analysis of different gene clusters shown in the heatmap.

**Figure S6. scMeT-seq dissects distinct cell states with defined metabolome and transcriptome.** (**A**) GO enrichment analysis of upregulated genes in State 3 shown in Data S2. (**B**) The normalized intensities of representative metabolites and the GSVA score of REACTOME GLYCOLYSIS on the pseudotime trajectory shown in Figure 4B. (**C**) The normalized intensities of representative metabolites and the GSVA score of REACTOME_THE_CITRIC_ACID_TCA_CYCLE_AND_RESPIRATORY_ELECTRON_TRANSPORT on the pseudotime trajectory shown in Figure 4B.

**Figure S7. Gene-metabolite correlation network constructed by scMeT-seq predicts biological functions of metabolites.** (**A**) Seahorse analysis of glycolysis of primary macrophages with indicated treatments for 72h. Bar graph showing the differential levels of basal glycolysis, compensatory glycolysis and the contribution of the glycolytic pathway to total extracellular acidification (% PER from glycolysis). (**B**) Venn plot showing the overlaps of downregulated or upregulated DEGs of indicated treatments.

**Table S1. List of metabolites obtained from the mass spectroscopy of the sorted sub-picoliter cytoplasm.**

| **Metabolites** | ***m/z*** | **Formula** | **Ion type** |
| --- | --- | --- | --- |
| Acetic acid | 59.0139 | C2H4O2 | [M-H]^-^ |
| Glyoxylic acid | 72.9931 | C2H2O3 | [M-H]^-^ |
| Lactaldehyde OR Propionic acid | 73.0295 | C3H6O2 | [M-H]^-^ |
| Glycine | 74.0248 | C2H5NO2 | [M-H]^-^ |
| Glycolic acid | 75.0088 | C2H4O3 | [M-H]^-^ |
| Crotonic acid | 85.0295 | C4H6O2 | [M-H]^-^ |
| Pyruvic acid | 87.0088 | C3H4O3 | [M-H]^-^ |
| Alanine OR Sarcosine OR Lactamide | 88.0404 | C3H7NO2 | [M-H]^-^ |
| Oxalate | 88.988 | C2H2O4 | [M-H]^-^ |
| Lactic acid | 89.0244 | C3H6O3 | [M-H]^-^ |
| Acetoacetic acid OR Succinic acid semialdehyde OR 2-Ketobutyric acid OR (S)-Methylmalonic acid semialdehyde OR 2-Methyl-3-oxopropanoic acid | 101.0244 | C4H6O3 | [M-H]^-^ |
| N-Ethylglycine OR 3-Aminobutanoic acid OR gamma-Aminobutyric acid | 102.0561 | C4H9NO2 | [M-H]^-^ |
| Malonic acid | 103.0037 | C3H4O4 | [M-H]^-^ |
| Cysteinyl | 103.0097 | C3H6NOS | [M-H]^-^ |
| L-Serine | 104.0353 | C3H7NO3 | [M-H]^-^ |
| 3-Mercaptopropanoic acid | 105.0016 | C3H6O2S | [M-H]^-^ |
| Glyceric acid | 105.0193 | C3H6O4 | [M-H]^-^ |
| Hypotaurine | 108.0125 | C2H7NO2S | [M-H]^-^ |
| Uracil | 111.02 | C4H4N2O2 | [M-H]^-^ |
| Acetylenedicarboxylic acid | 112.988 | C4H2O4 | [M-H]^-^ |
| L-Proline | 114.0561 | C5H9NO2 | [M-H]^-^ |
| L-Valine | 116.0717 | C5H11NO2 | [M-H]^-^ |
| Succinic acid | 117.0193 | C4H6O4 | [M-H]^-^ |
| L-Threonine | 118.051 | C4H9NO3 | [M-H]^-^ |
| Purine | 119.0363 | C5H4N4 | [M-H]^-^ |
| 2-Hydroxybenzaldehyde | 121.0295 | C7H6O2 | [M-H]^-^ |
| Nicotinic acid | 122.0248 | C6H5NO2 | [M-H]^-^ |
| Taurine | 124.0074 | C2H7NO3S | [M-H]^-^ |
| Ciliatine | 124.0169 | C2H8NO3P | [M-H]^-^ |
| 5-oxo-D-proline | 128.0353 | C5H6NO3 | [M-H]^-^ |
| Itaconic acid OR Mesaconic acid OR Glutaconic acid | 129.0193 | C5H6O4 | [M-H]^-^ |
| Ketoleucine | 129.0557 | C6H10O3 | [M-H]^-^ |
| Asparaginamide OR Creatine | 130.0622 | C4H9N3O2 | [M-H]^-^ |
| Leucine OR Isoleucine | 130.0874 | C6H13NO2 | [M-H]^-^ |
| Glutaric acid OR 2-Acetolactate OR Ethylmalonic acid | 131.035 | C5H8O4 | [M-H]^-^ |
| L-Asparagine OR Glycyl-glycine | 131.0462 | C4H8N2O3 | [M-H]^-^ |
| 2-Hydroxyisocaproic acid | 131.0714 | C6H12O3 | [M-H]^-^ |
| Aspartic acid | 132.0302 | C4H7NO4 | [M-H]^-^ |
| Malic acid | 133.0142 | C4H6O5 | [M-H]^-^ |
| Adenine | 134.0472 | C5H5N5 | [M-H]^-^ |
| L-Thyronine | 135.5428 | C15H15NO4 | [M-2H]^2-^ |
| 3-Nitrophenol | 138.0197 | C6H5NO3 | [M-H]^-^ |
| Phosphonoacetic acid | 138.9802 | C2H5O5P | [M-H]^-^ |
| O-Phosphoethanolamine | 140.0118 | C2H8NO4P | [M-H]^-^ |
| Caprylic acid | 143.1078 | C8H16O2 | [M-H]^-^ |
| Oxoglutaric acid | 145.0142 | C5H6O5 | [M-H]^-^ |
| Glutamine | 145.0619 | C5H10N2O3 | [M-H]^-^ |
| L-Glutamic acid | 146.0459 | C5H9NO4 | [M-H]^-^ |
| Nonanoate | 157.1234 | C9H18O2 | [M-H]^-^ |
| N-Methyllysine | 159.1139 | C7H16N2O2 | [M-H]^-^ |
| L-Phenylalanine | 164.0717 | C9H11NO2 | [M-H]^-^ |
| Phosphoenolpyruvic acid | 166.9751 | C3H5O6P | [M-H]^-^ |
| Glutathione thiol | 168.5207 | C10H17N3O6S2 | [M-2H]^2-^ |
| Glyceraldehyde 3-phosphate | 168.9907 | C3H7O6P | [M-H]^-^ |
| Glycerol 3-phosphate | 171.0064 | C3H9O6P | [M-H]^-^ |
| Decanoic acid | 171.1391 | C12H24O2 | [M-H]^-^ |
| L-Arginine | 173.1044 | C6H14N4O2 | [M-H]^-^ |
| N-Acetyl-L-aspartic acid | 174.0408 | C6H9NO5 | [M-H]^-^ |
| Ascorbic acid OR Diglyceride | 175.0248 | C6H8O6 | [M-H]^-^ |
| Fructose OR Glucose OR Galactose | 179.0561 | C6H12O6 | [M-H]^-^ |
| L-Tyrosine | 180.0666 | C9H11NO3 | [M-H]^-^ |
| Glyceraldehyde phosphate | 180.9544 | C3H3O7P | [M-H]^-^ |
| Phosphorylcholine | 182.0593 | C5H15NO4P | [M-H]^-^ |
| 3-Phosphoglyceric acid OR 2-Phosphoglyceric acid | 184.9857 | C3H7O7P | [M-H]^-^ |
| Citric acid or Isocitric acid | 191.0197 | C6H8O7 | [M-H]^-^ |
| D-Mannonic acid OR Gulonic acid OR Gluconic acid OR Galactonic acid | 195.051 | C6H12O7 | [M-H]^-^ |
| L-Dopa | 196.0615 | C9H11NO4 | [M-H]^-^ |
| Dodecanoic acid | 199.1704 | C12H24O2 | [M-H]^-^ |
| N-Acetyl-L-phenylalanine | 206.0823 | C11H13NO3 | [M-H]^-^ |
| Phosphocreatine | 210.0285 | C4H10N3O5P | [M-H]^-^ |
| N-Lactoyl ethanolamine phosphate | 212.0329 | C5H12NO6P | [M-H]^-^ |
| 5-Deoxyribose-1-phosphate OR Deoxyribose 5-monophosphate OR Deoxyribose 1-phosphate OR Deoxyribose 5-phosphate | 213.017 | C5H11O7P | [M-H]^-^ |
| GPEA | 214.0486 | C5H14NO6P | [M-H]^-^ |
| D-Ribulose 5-phosphate | 229.0119 | C5H11O8P | [M-H]^-^ |
| CTP | 240.485 | C9H16N3O14P3 | [M-2H]^2-^ |
| UTP | 240.977 | C9H15N2O15P3 | [M-2H]^2-^ |
| Cytidine | 242.0782 | C9H13N3O5 | [M-H]^-^ |
| Biotin | 243.0809 | C10H16N2O3S | [M-H]^-^ |
| Glycerophosphoglycerol | 245.0432 | C6H15O8P | [M-H]^-^ |
| Bisphenol S | 249.0227 | C12H10O4S | [M-H]^-^ |
| ATP | 252.4906 | C10H16N5O13P3 | [M-2H]^2-^ |
| Glucose 6-phosphate OR Glucose 1-phosphate OR myo-Inositol 1-phosphate OR myo-Inositol 6-phosphate | 259.0224 | C6H13O9P | [M-H]^-^ |
| Phosphogluconic acid | 275.0174 | C6H13O10P | [M-H]^-^ |
| UDP-D-Glucose OR UDP-Galactose | 282.0202 | C15H24N2O17P2 | [M-2H]^2-^ |
| Glutamine-glutamate | 290.0994 | C10H17N3O7 | [M-H]^-^ |
| Galactosyl 4-hydroxyproline OR 4-Hydroxyproline galactoside | 292.1038 | C11H19NO8 | [M-H]^-^ |
| Phenylalanylglutamic acid OR Hydroxyprolyl-Tyrosine | 293.1143 | C14H18N2O5 | [M-H]^-^ |
| UDP-GlcNAc | 302.5335 | C17H27N3O17P2 | [M-2H]^2-^ |
| CTMP | 303.0388 | C10H13N2O7P | [M-H]^-^ |
| GSSG | 305.0687 | C20H32N6O12S2 | [M-2H]^2-^ |
| GSH | 306.0765 | C10H17N3O6S | [M-H]^-^ |
| N-Acetylneuraminic acid OR Glucose-6-glutamic acid | 308.0987 | C11H19NO9 | [M-H]^-^ |
| TRP-Asp | 318.1095 | C15H17N3O5 | [M-H]^-^ |
| CMP | 322.0446 | C9H14N3O8P | [M-H]^-^ |
| UMP | 323.0286 | C9H13N2O9P | [M-H]^-^ |
| CAMP | 328.0452 | C10H12N5O6P | [M-H]^-^ |
| Glycerophosphoinositol | 333.0592 | C9H19O11P | [M-H]^-^ |
| AMP OR 3-AMP | 346.0558 | C10H14N5O7P | [M-H]^-^ |
| GMP OR 2-GMP OR 3-GMP | 362.0507 | C10H14N5O8P | [M-H]^-^ |
| S-Lactoylglutathione | 378.0977 | C13H21N3O8S | [M-H]^-^ |
| CDP | 402.0109 | C9H15N3O11P2 | [M-H]^-^ |
| UDP | 402.9949 | C9H14N2O12P2 | [M-H]^-^ |
| ADP | 426.0221 | C10H15N5O10P2 | [M-H]^-^ |
| Guanosine-3,5-Diphosphate | 442.0171 | C10H15N5O11P2 | [M-H]^-^ |
| UDP-D-galacturonic OR UDP-D-Glucuronate | 579.027 | C15H22N2O18P2 | [M-H]^-^ |
| NAD | 662.1018 | C21H27N7O14P2 | [M-H]^-^ |
| NADH | 664.1175 | C21H29N7O14P2 | [M-H]^-^ |

**Movie S1. (separate file)**

Real-time video showing that 9 μm opening capillary filled with SMART-seq2 lysis buffer approached the cell with the control of micromanipulator and aspirated cells with the application of an air pump device.

**Supporting Data**

**Data S1. (separate file)**

Differential expressed gene (DEG) analysis of single-cell transcriptomic data between indicated groups (scMeT-seq and whole-cell SMART-seq2; lactate+AZD3965 and lactate; lactate and control). Due to the small sample size in our study, genes with |LogFC|>0.25 and P<0.05 were considered as signature genes. Multiple testing corrections would significantly reduce the statistical power.

**Data S2. (separate file)**

Upregulated genes in different states (State1, 2 and 3) of macrophages shown in Figure 4B.

**Data S3. (separate file)**

GENIE3-inferring regulatory links (with the weight higher than 0.1) between metabolites and genes using the merged matrix of scMeT-seq.

**Data S4. (separate file)**

GENIE3-inferring regulatory links (with the weight higher than 0.1) between metabolites and genes using the merged matrix of scMeT-seq.

**Data S5. (separate file)**

DEG analysis of primary mouse macrophages between indicated groups in bulk RNA-seq.Lac: lactate; Lac_gly: co-treatment of lactate and glycerophosphoinositol; Lac_pho: co-treatment of lactate and phosphorylcholine; Lac_tau: co-treatment of lactate and taurine.
